# Endoscopic direct-vision therapy for acute uncomplicated appendicitis: a retrospective study

**DOI:** 10.3389/fmed.2026.1786918

**Published:** 2026-03-25

**Authors:** Li-peng Chen, Feng Zhou, Xian-jin Yu, Yu-liang Feng

**Affiliations:** Department of Gastroenterology, Zhejiang Hospital, Hangzhou, Zhejiang, China

**Keywords:** appendicitis, direct vision, endoscopic direct-vision appendicitis therapy, endoscopic retrograde appendicitis therapy, ultra-minimally invasive treatment

## Abstract

**Background:**

Acute uncomplicated appendicitis is common. Laparoscopic appendectomy (LA) is definitive but invasive, while endoscopic retrograde appendicitis therapy (ERAT) carries radiation risks. Endoscopic direct-vision appendicitis therapy (EDAT), an ultra-minimally invasive organ-preserving technique, lacks clarity on patient selection, long-term recurrence, and clinical applicability.

**Aim:**

To explore EDAT’s short-term efficacy, safety, and applicable population via comparison with LA, and contextualize it within existing evidence.

**Methods:**

A retrospective study (Dec 2023–Dec 2024, Zhejiang Hospital) included 41 EDAT and 46 LA patients. Clinical indicators, serum inflammatory factors (WBC, CRP), recurrence, and complications were compared; subgroup (gender/age) and correlation analyses were performed.

**Results:**

Baseline characteristics (sex, age, follow-up time) were balanced between the two groups (*P* > 0.05). Compared with LA group, EDAT group had significantly shorter operation time [(35.37 ± 10.48) min vs. (53.54 ± 7.91) min], faster abdominal pain relief [12.00 (12.00, 24.00) h vs. 60.00 (60.00, 72.00) h], earlier resumption of oral intake [6.00 (6.00, 12.00) h vs. 24.00 (12.00, 24.00) h], and better postoperative inflammatory control (postoperative WBC: 6.83 ± 1.39 vs. 7.62 ± 2.13 × 10^9^/L; postoperative CRP: 7.89 ± 2.98 vs. 19.90 ± 11.30 mg/L). The mean postoperative hospital stay in EDAT group was 3.00 (2.00, 3.50) days, and 3 patients (7.32%) developed recurrence during 1–13 months of follow-up (mean 7.88 ± 3.52 months). Subgroup analysis showed consistent efficacy of EDAT across genders and age groups.

**Conclusion:**

Endoscopic direct-vision appendicitis therapy is a feasible, safe ultra-minimally invasive alternative for selected patients, outperforming LA in symptom relief and inflammation control. However, single-center, small-sample, and short follow-up limit conclusions; large-scale prospective studies with 1–3 years of follow-up are needed.

## Introduction

Appendicitis is one of the most common causes of acute abdominal pain, with an incidence of approximately 7%–8% (1). For over a century, surgical appendectomy has been the first-line treatment for appendicitis. However, recent studies have suggested that surgical resection may be unnecessary for acute uncomplicated appendicitis (2, 3). Current research indicates that the appendix functions as a reservoir for gut microbiota and an important lymphoid organ, playing a critical role in immune regulation (4). Studies have shown that patients who undergo appendectomy have a significantly higher risk of colorectal cancer, cardiovascular disease, gallstones, and other conditions compared to the general population (5). In 2009, Professor Liu et al. (6) first proposed the concept of endoscopic retrograde appendicitis therapy (ERAT) for treating uncomplicated acute or chronic appendicitis of various etiologies. This technique not only preserves the appendix but also effectively resolves inflammation, marking a new era in appendicitis management. In recent years, non-operative management of acute uncomplicated appendicitis has gained increasing attention, primarily consisting of antibiotic therapy. Studies have shown that antibiotic treatment alone can achieve successful outcomes in a substantial proportion of patients, offering a non-invasive alternative to surgery (2, 3). However, such conservative approaches are associated with considerable recurrence rates, extended treatment durations, and variable efficacy. This underscores the need for minimally invasive interventions that preserve the appendix while providing immediate therapeutic effect. Endoscopic therapies, including ERAT and the more recent EDAT, have emerged as promising organ-preserving options within this landscape, aiming to combine the benefits of immediate source control with minimal invasiveness (6). It aligns with the “ultra-minimally invasive” concept proposed by Professor Linghu (7). However, traditional ERAT is performed under fluoroscopic guidance, which carries the risk of radiation exposure. Additionally, the limited visualization provided by fluoroscopy often makes it difficult to confirm complete removal of etiological factors (e.g., obstructing fecaliths), leading to short-term recurrence in some patients.

Endoscopic direct-vision appendicitis therapy (EDAT) involves inserting a subscope through the colonoscope biopsy channel to directly visualize the appendiceal lumen. This approach enables real-time observation, facilitates more precise operation, and avoids radiation exposure, achieving favorable clinical outcomes (8). A recent network meta-analysis by Wang et al. (9) highlighted the trade-offs in the management of uncomplicated appendicitis: appendectomy remains the most definitive option for preventing recurrence, while ERAT and similar techniques are associated with the lowest complication rates. This study aims to contextualize EDAT within this evidence framework, evaluate its short-term efficacy, and address practical considerations, such as institutional feasibility and global applicability, to inform clinical decision-making.

## Materials and methods

### General information

This was a retrospective comparative study involving patients hospitalized in the Department of Gastroenterology and Department of Surgery at Zhejiang Hospital from December 2023 to December 2024. Patients were divided into two groups: EDAT group (*n* = 41) who underwent endoscopic direct-vision therapy, and LA group (*n* = 46) who underwent laparoscopic appendectomy. All patients were fully informed of the two treatment options (LA and EDAT) with detailed explanations of the procedures, potential benefits, risks, and recovery processes, and made their own independent choices without coercion. Both groups were diagnosed with acute uncomplicated appendicitis, defined as appendicitis without signs of perforation, abscess, or diffuse peritonitis, confirmed by abdominal computed tomography (CT) or ultrasound with appendix diameter ≥ 6 mm, wall thickening (≥3 mm), and/or fecalith visualization (10). The Alvarado score (ranging 0–10) comprises six clinical and laboratory indicators: right lower quadrant pain (2 points), rebound tenderness (1 point), fever (1 point), nausea/vomiting (1 point), leukocytosis (2 points), and left shift of the white blood cell (WBC) count (2 points). A score ≥ 5 was considered supportive of a diagnosis of acute appendicitis (11).

Inclusion criteria (consistent for both groups): (1) Diagnosis of acute uncomplicated appendicitis confirmed by abdominal CT/ultrasound and Alvarado score 5–7 (moderate inflammation, excluding severe inflammation with Alvarado score ≥ 8) (11); (2) Appendix diameter 6–10 mm (mild-to-moderate dilation) without massive fecalith impaction (fecalith diameter < 5 mm) (10); (3) Age 18–75 years; (4) No contraindications to endoscopic or laparoscopic surgery; (5) No severe comorbidities (e.g., end-stage organ failure, coagulopathy) that may increase procedural risk; (6) The presence of fecaliths was assessed via preoperative imaging (CT/ultrasound) and recorded. In both groups, only patients with fecalith diameter < 5 mm were included to ensure feasibility of endoscopic removal in the EDAT group.

Exclusion criteria (consistent for both groups): (1) Complicated appendicitis (abdominal abscess, intestinal obstruction, diffuse peritonitis) (10); (2) Severe organ failure or dysfunction; (3) Psychiatric illness or inability to cooperate with the procedure; (4) Other causes of acute abdominal pain; (5) Pregnancy or lactation; (6) Appendix diameter > 10 mm or massive fecalith impaction (fecalith diameter ≥ 5 mm) predicted to be difficult for endoscopic removal; (7) History of abdominal surgery with severe adhesions; (8) To exclude catarrhal or mild self-limiting appendicitis, we included only patients with Alvarado scores of 5–7, indicating moderate inflammation, and imaging confirmation of appendiceal diameter ≥ 6 mm and wall thickening ≥ 3 mm. Patients with Alvarado scores < 5 or with only mild sonographic changes without clear inflammatory signs were not enrolled.

This study was approved by the Ethics Committee of Zhejiang Hospital (Approval No.: ZJHIRB-003K), with a waiver of informed consent due to the retrospective nature.

### Main drugs and instruments

1. EDAT group: Appendiceal irrigation was performed using 0.9% sodium chloride injection (Zhejiang Guoxing Pharmaceutical Co., Ltd., Hangzhou, China) and metronidazole sodium chloride injection (Hangzhou Minsheng Pharmaceutical Co., Ltd., Hangzhou, China). The tools used for appendiceal intubation included a high-frequency incision knife (4.9 F × 20 mm; Boston Scientific, Marlborough, MA, United States), a guidewire (0.035′′ D450; Changzhou Leao Medical Technology Co., Ltd., Changzhou, China), a disposable electronic appendiceal endoscope (subscope; Hangzhou Lesite Medical Technology Co., Ltd., Hangzhou, China), and the eyeMax system (Nanwei Medical Technology Co., Ltd., Nanjing, China). All procedures were performed using an Olympus CF-H290I electronic colonoscope (Tokyo, Japan). Metronidazole sodium chloride injection was selected for appendiceal irrigation due to its broad anaerobic coverage, which is relevant in appendicitis where polymicrobial infection often includes anaerobes. Although *Escherichia coli* is a common pathogen, anaerobic bacteria are frequently involved in appendiceal infections, and metronidazole provides effective coverage against them.
2. LA group: (1) Laparoscopic equipment: Olympus 30° laparoscope (Olympus Corporation, Tokyo, Japan), high-definition endoscopic imaging system (Karl Storz SE & Co., KG, Tuttlingen, Germany), carbon dioxide insufflator (Stryker Corporation, Kalamazoo, Michigan, USA, flow rate 0–30 L/min); (2) Surgical instruments: Disposable laparoscopic trocars (10 mm × 1, 5 mm × 2; Covidien LLC, Mansfield, Massachusetts, USA), laparoscopic scissors (Aesculap AG, Tuttlingen, Germany), ultrasonic scalpel (Ethicon Harmonic ACE, Ethicon Inc., Somerville, New Jersey, USA), laparoscopic grasper (Jiangsu Kanghui Medical Devices Co., Ltd., Huai’an, China), disposable endo-loop ligature (Medtronic plc, Dublin, Ireland), suction irrigation device (Shanghai Pudong Jinhuan Medical Equipment Co., Ltd., Shanghai, China).

### Treatment methods

1. EDAT group: All patients underwent EDAT within 12 h of admission. Preoperative bowel preparation followed conventional colonoscopy protocols, with patients receiving 3 L of polyethylene glycol. Under intravenous anesthesia, a colonoscopy with a clear cap was advanced to the ileocecal region to identify the appendiceal orifice. For patients with significant inflammation around the appendiceal orifice, intubation of the appendiceal lumen was facilitated using a high-frequency incision knife and guidewire, followed by advancement of the subscope through the colonoscope biopsy channel along the guidewire. In cases with only mild inflammation, direct subscope intubation was performed. In all cases, the subscope was advanced to the appendiceal fundus to visualize the lumen and mucosa and to assess for fecaliths, pus, and other pathological findings. Concurrent fecalith removal and irrigation were performed as needed. Irrigation began with 100 mL of metronidazole sodium chloride injection, followed by 0.9% sodium chloride injection until the effluent was clear and no residual fecaliths were observed. For large, endoscopically non-removable fecaliths, a plastic stent was placed to ensure drainage patency. All procedures were performed by three senior physicians with relevant technical expertise. Postoperative oral intake was permitted based on intraoperative luminal inflammation and abdominal pain relief. Discharge criteria included complete resolution of abdominal pain (Visual Analog Scale score = 0), normalization of WBC count (4–10 × 10^9^/L) and C-reactive protein (CRP) level (<10 mg/L), and the ability to tolerate oral intake without adverse reactions. Follow-up was conducted *via* telephone. For further technical details, readers are directed to a previously published EDAT protocol with accompanying video (12).
2. LA group: All patients underwent standard laparoscopic appendectomy under general anesthesia, performed by senior surgeons in the Department of Surgery, following conventional surgical protocols.

### Observation indicators

(1) Clinical characteristics: Sex, age, follow-up time; (2) Operation-related indicators: Operation time, postoperative abdominal pain relief time (VAS score ≤ 2), time to resume oral intake, hospital stay; (3) Serum inflammatory factors: WBC and CRP before treatment and 2 days after treatment; (4) Postoperative recurrence (defined as reoccurrence of acute appendicitis symptoms + elevated WBC/CRP + abdominal CT/ultrasound confirmation) and complications; (5) Resource utilization: Procedure duration, hospital stay cost (not fully recorded due to retrospective design, noted as a limitation).

### Statistical analysis

Data were analyzed using SPSS version 23.0 (IBM Corp., Armonk, NY, United States). Measurement data were expressed as mean ± standard deviation (x ± s) if normally distributed (Shapiro-Wilk test, *P* > 0.05) and compared using independent samples *t*-test; if abnormally distributed, expressed as median (interquartile range) [M(Q1,Q3)] and compared using Mann-Whitney U test. Categorical data were expressed as n (%) and compared using χ^2^ test. Intragroup comparisons of preoperative and postoperative indicators were performed using paired *t*-test or Wilcoxon signed-rank test. Correlation analysis between variables was performed using Pearson correlation (normally distributed data) or Spearman correlation (abnormally distributed data), with correlation coefficient r reported. A two-tailed *P* < 0.05 was considered statistically significant (*P < 0.05, **P < 0.01).

## Results

### Baseline characteristics and success rate

As shown in Table 1, there were no significant differences in sex, age, or follow-up time between EDAT group and LA group (all *P* > 0.05), indicating balanced baseline characteristics. The proportion of patients with preoperative imaging-confirmed fecaliths was 43.9% (18/41) in the EDAT group and 43.5% (20/46) in the LA group.

**TABLE 1:** Clinical characteristics (EDAT = 41, LA = 46).

Variables	EDAT group	LA group	Test statistic	*P*-value
Sex, male, *n* (%)	19 (46.3%)	24 (52.2%)	χ^2^ = 0.295	0.669
Age, year, mean ± SD	43.41 ± 15.01	44.09 ± 13.46	*t* = −0.220	0.826
Alvarado score, median (IQR)	6.00 (5.00, 6.00)	6.00 (5.00, 7.00)	χ^2^ = 0.859	0.651
Operation time, min, mean ± SD	35.37 ± 10.48	53.54 ± 7.91	*t* = −9.189	<0.001
Postoperative abdominal pain relief time, h, median (IQR)	12.00 (12.00, 24.00)	60.00 (60.00, 72.00)	*U* = 6.00	<0.001
Time to oral intake, h, median (IQR)	6.00 (6.00, 12.00)	24.00 (12.00, 24.00)	*U* = 255.000	<0.001
Hospital stay days, median (IQR)	3.00 (2.00, 3.50)	5.00 (5.00, 6.00)	*U* = 61.000	<0.001
Follow up time, months, mean ± SD	7.88 ± 3.52	8.70 ± 2.33	*t* = −1.216	0.212

EDAT, endoscopic direct-vision appendicitis therapy; LA, laparoscopic appendectomy; IQR, interquartile range; SD, standard deviation.

Regarding operation-related indicators, EDAT group had significantly shorter operation time, faster abdominal pain relief, earlier resumption of oral intake and shorter hospital stay compared with LA group (all *P* < 0.001).

### Perioperative outcomes

As shown in Table 2, preoperative WBC was comparable between the two groups (*P* = 0.423), while preoperative CRP in EDAT group was significantly lower than that in LA group (*P* = 0.002), possibly reflecting milder baseline inflammation in EDAT-selected patients. After treatment, both groups showed significant reductions in WBC and CRP (all *P* < 0.001). Postoperatively, WBC and CRP in EDAT group were significantly lower than those in LA group (*P* = 0.043 and *P* < 0.001, respectively), indicating better inflammatory control.

**TABLE 2:** Serum inflammatory factors.

Variables	EDAT	LA	*t*-value	*P*-value
WBC (10^9^/L), mean ± SD				
Preoperative	14.82 ± 2.03	15.24 ± 2.78	−0.805	0.423
Postoperative	6.83 ± 1.39	7.62 ± 2.13	−2.061	0.043
t/Z value	*t* = 31.406	*Z* = −5.905	–	–
*P*-value	<0.001	<0.001	–	–
CRP (mg/L)				
Preoperative	60.97 ± 24.32	76.95 ± 21.45	−3.258	0.002
Postoperative	7.89 ± 2.98	19.90 ± 11.30	−6.938	<0.001
t/Z value	*Z* = −5.579	*t* = 28.488	–	–
*P*-value	<0.001	<0.001	–	–

CRP, C-reactive protein; WBC, white blood cell.

### Postoperative recurrence

During a mean follow-up of 7.88 ± 3.52 months (range 1–13 months), 3 patients (7.32%) in EDAT group developed recurrent acute uncomplicated appendicitis: 1 case at 1 month (with residual fecalith), 1 case at 3 months, and 1 case at 6 months. All 3 patients declined repeat EDAT and underwent laparoscopic appendectomy with no complications. The remaining 38 patients experienced no recurrence, resulting in an overall recurrence rate of 7.32%. No recurrence was observed in LA group during follow-up. No intraoperative or psotoperative complications (bleeding, perforation) occurred in either group.

#### Subgroup analysis and correlation analysis

As shown in Table 3, subgroup analysis of EDAT group by gender and age revealed significant reductions in WBC and CRP after treatment in all subgroups (all *P* < 0.001), indicating consistent efficacy. Correlation analysis (Table 4) showed that preoperative WBC and CRP were strongly positively correlated with postoperative hospital stay (*r* = 0.829, *P* < 0.001; *r* = 0.742, *P* < 0.001) and postoperative CRP (*r* = 0.711, *P* < 0.001; *r* = 0.844, *P* < 0.001), suggesting that severe preoperative inflammation is associated with longer recovery.

**TABLE 3:** Serum inflammatory factors subgroup stratified analysis for EDAT.

Variables	Preoperative	Postoperative	t/Z value	*P*-value
Male				
WBC (10^9^/L)	15.18 ± 2.42	7.09 ± 1.65	19.263	<0.001
CRP (mg/L)	62.45 (49.21, 83.77)	7.69 (6.87, 10.07)	−3.823	<0.001
Female				
WBC (10^9^/L)	14.52 ± 1.62	6.61 ± 1.10	25.191	<0.001
CRP (mg/L)	56.20 ± 14.33	7.14 ± 1.53	16.684	<0.001
Age < 45 years				
WBC (10^9^/L)	14.23 (12.89, 15.11)	6.91 (5.77, 8.02)	−4.198	<0.001
CRP (mg/L)	55.88 (41.26, 68.21)	7.69 (5.71, 9.07)	−4.198	<0.001
Age ≥ 45 years				
WBC (10^9^/L)	15.36 ± 1.32	7.07 ± 1.25	36.306	<0.001
CRP (mg/L)	60.93 (50.77, 71.72)	7.34 (6.82, 8.45)	−3.724	<0.001

CRP, C-reactive protein; WBC, white blood cell.

**TABLE 4:** Correlation analysis between preoperative WBC, preoperative CRP, operative time and postoperative indicators (r).

Variables	Preoperative WBC	Preoperative CRP	Operation time
Postoperative abdominal pain relief time	0.311*	0.304	0.453**
Time to oral intake	0.502**	0.555**	0.521**
Hospital stay	0.829**	0.742**	0.339*
Follow up time	−0.276	−0.354*	−0.039
Postoperative WBC	0.603**	0.480**	0.206
Postoperative CRP	0.711**	0.844**	0.397*

CRP, C-reactive protein; WBC, white blood cell.

*P < 0.05,

**P < 0.01.

To evaluate whether baseline disease severity influenced treatment outcomes, we performed a subgroup analysis stratifying patients by Alvarado score into mild (score = 5) and moderate-to-severe (score = 6–7) groups (Table 5). In both subgroups, EDAT demonstrated significant advantages over LA, including shorter hospital stay, faster pain relief, and lower postoperative CRP (all *P* < 0.05). Postoperative WBC was lower in the EDAT group in both subgroups, but the differences were not statistically significant (both *P* > 0.05).

**TABLE 5:** Subgroup analysis based on Alvarado score: comparison of clinical outcomes between EDAT and LA.

Stratification standard	Group	Postoperative WBC (10^9^/L)	Postoperative CRP (mg/L)	Postoperative abdominal pain relief time (hour)	Hospital stay (day)
Mild group (Alvarado score = 5)	EDAT (*n* = 11)	7.09 ± 1.51	9.43 ± 4.63	24.00 (12.00, 24.00)	3.00 (2.00, 4.00)
	LA (*n* = 15)	8.06 ± 2.03	18.85 ± 11.73	72.00 (60.00, 84.00)	6.00 (5.00, 7.00)
	*P*	0.175	0.019	<0.001	<0.001
Moderate-to-severe group (Alvarado score = 6–7)	EDAT (*n* = 30)	6.74 ± 1.36	7.33 ± 1.91	12.00 (10.50, 24.00)	3.00 (2.00, 3.00)
	LA (*n* = 31)	7.41 ± 2.17	20.40 ± 11.25	60.00 (48.00, 72.00)	5.00 (5.00, 6.00)
	*P*	0.155	<0.001	<0.001	<0.001

*P*-values are for comparisons between EDAT and LA within each subgroup. Continuous variables were compared using the *t*-test based on the normality of distribution.

## Discussion

The pathogenesis of acute appendicitis primarily involves luminal obstruction followed by secondary bacterial infection. Since the first appendectomy was described in 1891, surgical removal of the appendix has remained the gold standard (13). Although LA has largely replaced open surgery, it is still associated with a substantial negative appendectomy rate (9%–20%) (14) and significant complications, including incisional infection, abdominal abscess, bleeding, intestinal obstruction, and fistula formation (15, 16). International consensus now emphasizes minimizing negative appendectomies without increasing perforation risk due to delayed diagnosis (17), prompting reevaluation of treatment strategies.

Data from the United States National Inpatient Sample indicate that non-surgical management of appendicitis increased between 2010 and 2014 compared to the previous decade (18). Similarly, a large-scale multicenter study (43 centers) conducted in China in 2017 reported a 21.2% rate of conservative treatment with a 98.6% success rate (19), indicating favorable long-term outcomes of non-surgical management of acute uncomplicated appendicitis (20). However, antibiotic therapy alone is limited by lower cure rates, prolonged treatment, variable efficacy, and high recurrence rates (21), making it less preferable for many patients.

Inspired by ERCP, Professor Liu Bing introduced ERAT in 2012, which relieves luminal obstruction and eliminates infection *via* appendiceal irrigation and stenting (22). Subsequent studies in China have confirmed that ERAT, when combined with antibiotic therapy, achieves high intubation success rates, low complication rates, and good clinical efficacy, while colonoscopy improves the identification of underlying etiological factors (23).

Endoscopic retrograde appendicitis therapy preserves the appendix by relieving obstruction through natural luminal access–using intubation, irrigation, and drainage–to rapidly reduce intraluminal pressure, pain, and inflammation (6, 24). However, traditional ERAT relies on fluoroscopic contrast imaging, which requires specialized equipment; involves complex operational steps; and carries risks of contrast allergy, residual contrast retention, and radiation exposure–concerns that are particularly significant for pregnant women and children. EDAT addresses these limitations by using a subscope for real-time visualization, targeted irrigation, and lithotripsy when needed, thereby avoiding the need for radiation and contrast. This approach eliminates dependence on fluoroscopy, enables comprehensive assessment of the appendiceal lumen, and has shown advantages over traditional ERAT, including shorter operation times and lower recurrence rates (24), consistent with our findings. Additionally, endoscopic management of incarcerated appendicitis has been reported (25), suggesting expanding clinical applications for EDAT.

In the present cohort, the prevalence of fecaliths was comparable between the two groups. Although preoperative CRP levels were lower in the EDAT group–suggesting potentially milder baseline inflammation–both groups met the same inclusion criteria, and postoperative inflammatory control remained superior in the EDAT group.

We acknowledge that preoperative CRP was lower in the EDAT group (*P* = 0.002), suggesting potential selection bias. To address this, we performed subgroup analysis by Alvarado score (Table 5). The results showed that EDAT consistently outperformed LA in both mild and moderate-to-severe subgroups (all *P* < 0.05), demonstrating that the benefits of EDAT are independent of baseline disease severity. Although EDAT presents distinct clinical advantages, its potential adverse events still require careful attention. For patients who develop recurrent appendicitis after EDAT and eventually undergo LA, the underlying causes for such a treatment conversion merit further investigation. Residual fecaliths can act as a persistent inflammatory focus, triggering repeated inflammation and luminal obstruction that ultimately necessitate surgery. Even when fecaliths are completely removed and thorough irrigation is performed, recurrence may still occur. This could be due to undetectable anatomical abnormalities of the appendiceal lumen, such as intrinsic stenosis, or incomplete resolution of mucosal inflammation, both of which can lead to re-obstruction and impaired drainage.

Patients at higher risk of postoperative recurrence requiring surgical intervention may include those with large, recalcitrant fecaliths that cannot be fully cleared and in whom stenting fails to ensure adequate drainage. Similarly, individuals with severe and extensive mucosal congestion and edema around the appendiceal orifice and lumen may face challenges in maintaining luminal patency after endoscopic treatment, predisposing them to recurrent inflammation. Moreover, patients with markedly elevated preoperative inflammatory markers (e.g., WBC and CRP levels) indicating severe infection may have more profound appendiceal wall damage, which could impair tissue repair following endoscopic management and increase the risk of recurrence, requiring surgery.

Nevertheless, EDAT remains a preferable and effective minimally invasive treatment option. Compared to LA, EDAT for acute uncomplicated appendicitis offers shorter operation times, reduced hospital stays, and better inflammatory control, aligning with the principles of ultra-minimally invasive, trauma-reducing intervention.

This technology is relatively new, and several limitations should be acknowledged. The retrospective study design, small sample size, and short follow-up period restrict the robustness and generalizability of the findings. Potential selection bias cannot be excluded: although patients independently chose between EDAT and LA after full informed consent, inherent differences in patient preferences (e.g., inclination toward organ preservation vs. definitive surgical treatment) or subtle variations in baseline inflammation severity (e.g., preoperative CRP was lower in the EDAT group, *P* = 0.002) may have influenced the observed outcomes. To confirm the clinical advantages suggested in this study, larger, multicenter randomized controlled trials are essential. Future prospective studies with extended follow-up (1–3 years) are needed to verify the long-term safety, durability, and recurrence profile of EDAT. Additionally, efforts should focus on establishing standardized procedural protocols, structured training programs, and accessible technical resources (e.g., video demonstrations, step-by-step guidelines) to promote broader dissemination of expertise as the technique continues to evolve.

In conclusion, for selected patients with acute uncomplicated appendicitis, EDAT shows short-term advantages over LA–including shorter operation time, faster postoperative recovery (quicker pain relief, earlier oral intake, and shorter hospital stay), and better inflammatory control (lower postoperative WBC and CRP). However, due to the retrospective design, small sample size, and short follow-up, its long-term recurrence risk and generalizability need confirmation by larger prospective studies.

## Data Availability

The raw data supporting the conclusions of this article will be made available by the authors, without undue reservation.
